# Effect of the Polysaccharide Extract from the Edible Mushroom *Pleurotus ostreatus* against Infectious Bursal Disease Virus

**DOI:** 10.3390/ijms10083616

**Published:** 2009-08-18

**Authors:** Mircea Selegean, Mihai V. Putz, Tatiana Rugea

**Affiliations:** 1 Laboratory of Plant Biotechnology, Biology Department, West University of Timişoara, Pestalozzi Str. 16, RO-300115, Romania; 2 Laboratory of Computational and Structural Physical Chemistry, Chemistry Department, West University of Timişoara, Pestalozzi Str. 16, RO-300115, Romania; 3 Laboratory of Virology, National Agency for Sanitary Veterinary and Food Safety, Timişoara Branch, Martir Caceu Str. 4, Timişoara, Romania

**Keywords:** extracellular fraction, Gumboro disease, BIAVAC & BIAROMVAC vaccines, ReBiAc principles

## Abstract

The polysaccharide-containing extracellular fractions (EFs) of the edible mushroom *Pleurotus ostreatus* have immunomodulating effects. Being aware of these therapeutic effects of mushroom extracts, we have investigated the synergistic relations between these extracts and BIAVAC and BIAROMVAC vaccines. These vaccines target the stimulation of the immune system in commercial poultry, which are extremely vulnerable in the first days of their lives. By administrating EF with polysaccharides from *P. ostreatus* to unvaccinated broilers we have noticed slow stimulation of maternal antibodies against infectious bursal disease (IBD) starting from four weeks post hatching. For the broilers vaccinated with BIAVAC and BIAROMVAC vaccines a low to almost complete lack of IBD maternal antibodies has been recorded. By adding 5% and 15% EF in the water intake, as compared to the reaction of the immune system in the previous experiment, the level of IBD antibodies was increased. This has led us to believe that by using this combination of BIAVAC and BIAROMVAC vaccine and EF from *P. ostreatus* we can obtain good results in stimulating the production of IBD antibodies in the period of the chicken first days of life, which are critical to broilers’ survival. This can be rationalized by the newly proposed reactivity biological activity (ReBiAc) principles by examining the parabolic relationship between EF administration and recorded biological activity.

## 1. Introduction

Traditionally, many types of mushrooms have been used as Traditional Chinese Medicine, as well as functional foods in Japan and other Asian countries. Edible mushrooms have beneficial health effects and in the treatment of some diseases thanks to their immunomodulatory, anti-neoplastic, anti-hyperglycemic and lipid-reducing properties [1,2]. In this way, water extracts of the Shiitake mushroom (*Lentinus edodes*), for example, have been shown to prevent tumors in mice. Mushroom (*P. ostreatus*) polysaccharides especially the high-molecular-weight β-d-glucan, have been considered to have anti-cancer activity [3]. These mushroom polysaccharides also exhibited direct inhibitory effects on cancer cell growth by modulating cell-cycle progression and inducing apoptosis [3–5].

Polysaccharides represent a structurally diverse class of macromolecules of relatively widespread occurrence in Nature. The biological activities of polysaccharides have attracted more and more attention in biochemistry and medicine. Many immunomodulation and antitumoral polysaccharides were isolated from mushrooms and extensively studied in Japan, China, and Europe [3].

Many polysaccharides purified from mushroom fungi are either homoglycans or heteroglycans, while others mostly bind to protein residues as polysaccharide-protein complexes. The fungal antitumor polysaccharides are mainly present as glucans with different types of glycosidic linkages, but some are true heteroglycans. The main source of immunomodulation and antitumor polysaccharides appears to be related to fungal cell walls which consist of polysaccharides such as chitin, cellulose, (1→3),(1→6)-β-glucans and (1→3)-α-glucans or polysaccharide-protein complexes such as galactomannan-protein or glucuromannan-protein. Several potent antitumor polysaccharide-protein complexes have been purified from fruiting bodies of a Chinese edible mushroom, *P. sajor caju*. The polysaccharide with strong antitumor activity isolated from *P. ostreatus* is a highly branched (1→3)-β-glucan having an average structure represented by a pentasaccharide segment consisting of one nonreducing terminus, on 3,6-*O*-substituted, and three 3-mono-*O*-substituted β-glucopyranosyl side chains [3].

It is known that an unbalanced immunitory system provides the premise for a great number of diseases in both humans and animals. Infectious bursal disease (IBD) is caused by a virus that is a member of the genus Avibirnavirus of the family Birnaviridae. According to the virus virulence and pathogenicity, IBD causes more severe or less severe lesions on the bursa of Fabricius and other organs such as spleen, thymus and kidneys, and may induce immunosuppression and mortality in birds. Infectious bursal disease virus (IBDV) causes clinical disease of considerable economic importance in chickens, usually between 3 and 6 weeks of age or possibly older, following infection with very virulent strains. Numerous vaccines and vaccination programs have been studied and proposed worldwide [6–8]. The effect of polysaccharides present in EF extracts from the edible mushroom *P. ostreatus* are investigated here with emphasis on the specific response of the chickens’ immunitary system. The following points have been investigated: (1) the effect of EF on unvaccinated chickens, (2) the effect of EF on broilers vaccinated with BIAVAC vaccine, (3) the effect of EF on broilers vaccinated with BIAROMVAC vaccine and (4) the parabolic reactivity analysis of biological activity.

By comparing the results, we have showed that EF from the edible mushroom *P. ostreatus* helps BIAVAC and BIAROMVAC vaccines in stimulating the immune system against IBDV during the critical first two weeks post hatching.

## 2. Results and Discussion

### 2.1. The effect of EF on unvaccinated chickens

**Figure 1.**
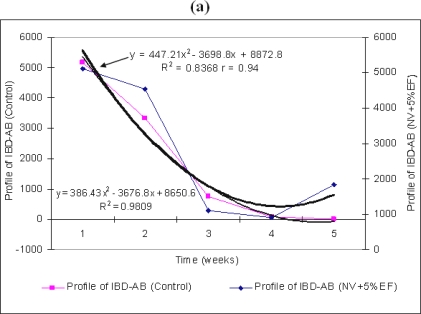

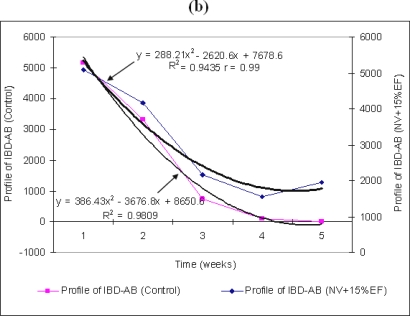
The effect of different concentrations of EF from *P. ostreatus* upon the maternal IBD–AB titer for unvaccinated chicken. Thin trend line —: the titer of maternal IBD–AB (*Control*); Thick trend line 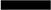: the titer of maternal IBD–AB in un-vaccinated chicken NV + 5% (a) and 15% EF (b). Weeks mean weeks post hatch.

In our study, we have followed the effect of EF from the edible mushroom *P. ostreatus* on broiler chickens which have not been vaccinated with BIAVAC and BIAROMVAC vaccines (Figure 1). In this case the maternal titers of infectious bursal disease virus antibodies (IBD–AB) (control) are seen to decrease, being at the minimum level (96) in four weeks post hatching (Figure 1a). This antibody titer (96) was below the estimated cut-off level in this enzyme–linked immunosorbent assay (ELISA) system.

By analyzing the variation (Table 1) we can observe significant differences (*p*<0.001) in the change of IBD–AB maternal titer. The variation coefficient is low (5.97%) in the first week of chicken life and high (20.37%) in week four.

The *F*-ratio, which in this case equals 3152.98, is a ratio of the between-group estimate to the within-group estimate. Since the *P*-value of the *F*-test is less than 0.05, there is a statistically significant difference between the means of the five weeks at the 95.0% confidence level. If we compare the distribution of maternal antibodies within each group during one week, but also between weeks, we can notice a significant difference (Table 2).

The difference between the antibodies titer averages in weeks four and five is not significant. Yet, by adding 5% EF in the water intake, we can notice a stabilization of the titer level for the stimulation of production of maternal IBD–AB, starting with the first days of week five (*p<*0.0001). The standard deviation from the average of the maternal IBD–AB titer value is high in the mid-period of our experiment (21.26% in week three) and low (0.67%) in the last week (Table 1).

The differences between the antibody titer averages between groups are significant, but those between the values of weeks three and four are insignificant. They are significant, but negative, between the average values of weeks three and five (−741.95) and between weeks four and five (−944.0) (Table 2). This last aspect has been shown by stimulation of antibodies production (Figure 1a) and is statistically explained by the chicken positive reaction (r=0.94) to EF action for IBD–AB stimulation and a homogeneity of broilers answers (biological activity) in IBD–AB production.

By supplementing the water intake with 15% EF from *P. ostreatus* (Figure 1b), standards deviations are low at the beginning of the experiment (3.68%) and towards the end of it (7.19% and 7.63%). The differences between antibodies titre averages between all averages antibodies groups are significant. A positive ratio (r=0.99) has been established between the two variables, EF and the capacity of chicken bodies to produce antibodies (Figure 1b). The unvaccinated broilers + 5% EF have shown a better global behaviour, whose average of variation coefficient has been 9.56%, respect to both the unvaccinated broilers + 15% EF with an average of 8.65% and those unvaccinated without EF, which show an average of about 8.106%.

### 2.2. The effect of EF on broiler vaccination with BIAVAC vaccine

**Figure 2.**
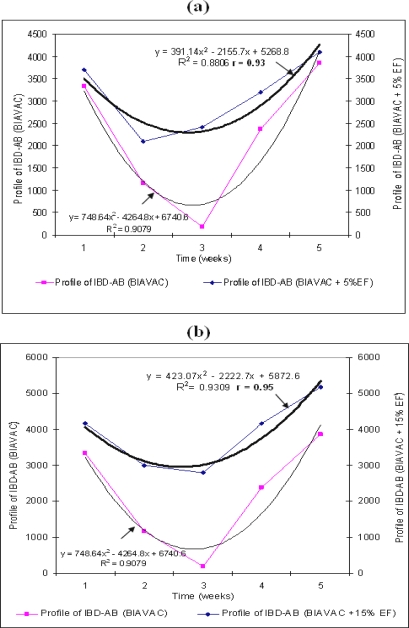
The effect of different concentration of EF from *P. ostreatus* upon maternal IBD–AB titre to broilers vaccinated with BIAVAC. Thin trend line —: the titre of maternal IBD–AB (BIAVAC); Thick trend line 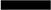: the titre of maternal IBD–AB to broilers with BIAVAC + 5% (a) and BIAVAC + 15% EF(b). Weeks mean weeks post hatch, with the vaccination at the end of week 1.

In this experimental group, we have followed the change of IBD–AB antibodies in broilers vaccinated with BIAVAC and treated with EF. Thus, all broilers, although vaccinated with BIAVAC, without EF treatment, have shown a decrease of the level of maternal IBD–AB antibodies. The lowest antibody titre (185) was below the estimated cut-off level in this ELISA system. The production of antibodies increased starting with week 4 (*p<*0.0001; Figure 2a; Table 3).

The final level of this biosynthesis of antibodies is above the value of the titre on the 1^st^ day. By treating the intake water with 5% EF, the values of IBD–AB titre are maintained at a relatively high level (*p*<0.0001), with slightly high indicators of variation coefficient, at the beginning and at the end of the treatment. We can notice (Figure 2a; Table 3) the action of EF in synergism with BIAVAC vaccine upon the production of antibodies (r=0.93). The standard deviations are significant when 15% EF was administered (*p*<0.0001; Figure 2b, Table 3).

By a multiple comparison between the pairs means of IBD–AB: 1–2, 1–3 and 1–4 which correspond to the immunosuppressive period (weeks 1 and 3), we can notice significant positive differences with the BIAVAC experiment. Starting with week 4, between the pairs means of IBD–AB, we can notice significant negative differences (Table 4). In this 2^nd^ half of the experimental period, the production of IBD–AB antibodies can be noticed, with a high level (3859.00), which is above the level of the 1^st^ day of post hatching. In the next experimental variants of the broilers (BIAVAC + 5% EF and BIAVAC + 15% EF), between the pairs means of IBD–AB we can notice significant differences of value, as compared to BIAVAC variants at the 95.0% confidence level (Table 4).

The averages of variations are relatively homogenous, 11.63% (BIAVAC non-EF), 14.76% (BIAVAC + 5% EF) and 13.87% (BIAVAC + 15% EF), while the correlation coefficients between the variables of the system are positive, r=0.93 (BIAVAC + 5% EF) and r=0.95 (BIAVAC + 15% EF) (Figures 2a,b).

### 2.3. The effect of EF on broilers vaccinated with BIAROMVAC vaccine

**Figure 3.**
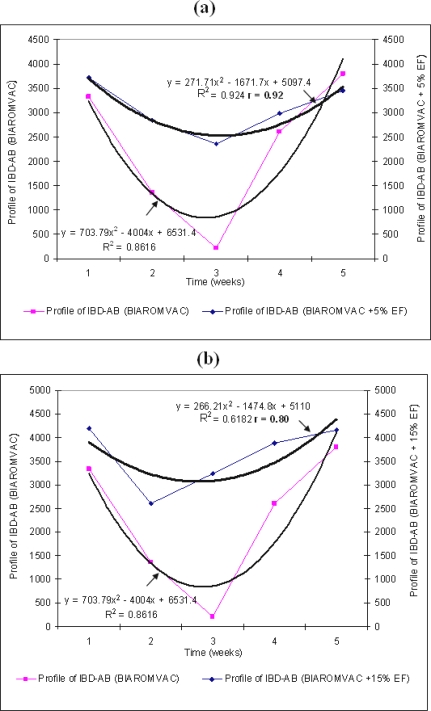
The effect of different concentrations of EF from *P. ostreatus* upon maternal IBD–AB titer in broilers vaccinated with BIAROMVAC vaccine. Thin trend line —: the titre of maternal IBD–AB (BIAROMVAC); Thick trend line 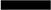: the titre of maternal IBD–AB in broiler BIAROMVAC + 5% (a) and BIAROMVAC + 15% EF (b). Weeks mean weeks post hatch, with the vaccination at the end of week 1.

In this experiment, we have followed the production of IBD–AB antibodies in non-vaccinated chickens, vaccinated with BIAROMVAC, as compared to the ones under BIAROMVAC + 5% EF treatment and BIAROMVAC + 15% EF treatment, respectively. Thus, in chickens only vaccinated with BIAROMVAC vaccine, (like BIAVAC vaccine for the previous experiment), we can notice the temporary suppressive action of maternal IBD–AB antibodies production, in the first two weeks post-vaccinated inoculation (Figures 3a,b). All broilers had a maternal IBD–AB titer of 217 in the ELISA. These antibodies were below the estimated cut-off level in this ELISA. In the experimental groups, to which we have added 5% and 15% EF, respectively, all along the duration of the experiment, the antibodies level remained high, with a slow decrease at the beginning of the treatment (Figure 3, Table 5; *p*<0.0001).

We have noticed a better response in broilers treated with BIAROMVAC + 5%EF, whose average variation was 12.85%, as compared to the treatments BIAROMVAC without EF and BIAROMVAC + 15% EF, with averages of 14.39 and 14.02%, respectively. By comparison, the all pairs means of maternal IBD–AB have shown significant differences at 95.0% confidence level (Table 6), except for one case (2–4) of the treatment with BIAROMVAC + 5% EF and two cases (1–4 and 1–5) of the treatment with BIAROMVAC + 15% EF. The correlation coefficient between the system variables are positive (r=0.92 and respectively, r=0.80) (Figures 3a,b).

The real guideline basis of this experiment relies on the immunosuppressive effects in non-vaccinated chickens by BIAVAC, BIAROMVAC vaccines, and other IBDV vaccines in the first critical days of broilers life [9,10]. The field and clinical observations have shown differences in response of different lines genetic backgrounds upon the influence of some IBDV pathogenesis upon maternal antibodies. Also, from economical reasons, the studies are worth initiated and continued since the production of broilers decreases because of maternal antibodies decrease in progenies (Tables 1, 3, 5). Another reason of these studies reflects the fact that not much is known as far the immunosuppressive abilities of IBD vaccines in commercial broilers is concerned [11–13]. In the present work, we compared immuno-modulatory effects of different concentrations of EF with polysaccharides extracted from *P. ostreatus* with, but also without BIAVAC and BIAROMVAC vaccines, on commercial broilers. Our study proves the immunomodulatory ability of EF with polysaccharides extracted from *P. ostreatus* together with BIAVAC and BIAROMVAC vaccines, towards the stimulation of IBD–AB antibodies production.

There are studies which demonstrate, to a certain degree of certitude, the existence of some connections between IBDV–induction of immunosuppression and the severe injuries which appear in bursa of Fabricius. Even more, there have been situations, even as hypothesis, of association of IBDV with B cells, macrophages and follicular dendritic cells in bursa and spleen [8]. Other fundamental studies mention that these IBD–AB vaccines may induce suppressive secondary effects in the immune system, such as systemic T cell activity. Thus, the macrophages may induce the release of cytokines and nitric oxide which can suppress T cell activity. Another hypothesis is that the suppression of T helper cell activity might favour to reduce IBD–AB antibodies response in broilers [14]. Also in the cases of our research, we have noticed such a temporary suppression of IBD–AB antibodies in all experiments in broilers (Figure 1–3), when we have used BIAVAC and BIAROMVAC vaccines. By supplementing the water intake with EF which has polysaccharides extracted from *P. ostreatus* we have noticed a stimulation of IBD–AB antibodies production in all experimental groups. Scientific data show that substances from edible mushrooms are responsible for imunodulating effects. These substances are β-d-glucan polysaccharides. The β-d-glucans are a heterogeneous group of glucose polymers. These polysaccharides are the major cells wall structural components found in fungi and also present in plant and some bacteria. Studies with β-d-glucan from *P. ostratus* or from *L. edodes*, have shown several immuno-modulatory properties which include increase of T-cells activity, restoration of T-helper cells suppression, induction of cytotoxic activity of peritoneal macrophages. The β-glucans bind to receptors on cells membranes of macrophages, neutrophils, natural killer cells, T-cells dendritic cells, fibroblast and vascular endothelial cells. The molecular structure of these substances from fungi influences the affinity to receptors and the stimulation of the immunomdulator system [15,16].

### 2.4. Reactivity analysis of the parabolic biological activity relationship

The results of previous sections opens the possibility for further systematic treatment of IBD–AB activity (*y*) through employing the fitted response curves of Figures 1–3 beyond the statistical correlation factor and fitted parameters.

Actually, since the reported ANOVA analysis in Figures 1–3 unfolds under the parabolic form:

(1) $$y=y_{0}-ax+bx^{2}$$

it readily reminding of the chemical reactivity law modeling the (molecular) systems’ energy change with the number of donated/accepted electrons Δ*N* :

(2) $$E=E_{equilibrium}-\chi \Delta N+\eta {\left(\Delta N\right)}^{2}$$

through the electronegativity *χ* and chemical hardness *η* indices:

(3a) $$\chi =\frac{IP+EA}{2}$$

(3b) $$\eta =\frac{IP-EA}{2}$$

in terms of ionization potential (*IP*) and electron affinity (*EA*), respectively [17].

Now, the idea is to rearrange Equation (1) under the equivalent form given by Equation (2) that will reveal us how the fitting coefficients of Equation (1) will correspond to the reactivity indices in Equation (2). Yet, such transformation is not direct, but upon considering appropriate operations. As such, the first step regards the translation of the form given by Equation (1) into a parabolic form centred on its optimum values, either for dependent *Y_opt_* and independent *X_opt_* variables:

(4) $$Y=Y_{opt}-\alpha \left(x-X_{opt}\right)+\beta {\left(x-X_{opt}\right)}^{2}$$

while the optimum expression are found by the variation of Equation (1):

(5) $$X_{opt}:{\frac{\partial y}{\partial x}|}_{x=X_{opt}}=0,Y_{opt}=y\left(X_{opt}\right)$$

or, analytically as:

(6) $$X_{opt}=\frac{a}{2b},Y_{opt}=y_{0}-\frac{a^{2}}{4b}$$

Then, in order for coefficients *α* and *β* to play the “reactive” role in Equation (4) as electronegativity and chemical hardness do in Equation (2), respectively, they have to be considered with a similar expression as that of Equation (3), namely:

(7a) $$\alpha =\frac{Y^{-}+Y^{+}}{2},$$

(7b) $$\beta =\frac{Y^{-}-Y^{+}}{2},$$

however, linked with the input equation (1) by means of assignments:

(8a) $$Y^{-}=y\left(x\to -1;y_{0}\to Y_{opt}\right),$$

(8b) $$Y^{+}=y\left(x\to +1;y_{0}\to Y_{opt}\right),$$

thus providing the results:

(9a) $$\alpha =Y_{opt}+b=y_{0}-\frac{a^{2}}{4b}+b,$$

(9b) β=a.

Now, paralleling the chemical reactivity principles of electronegativity and chemical hardness [17] to the coefficients *α* and *β* of Equation (4), respectively, one can infer the hierarchies among two biological-chemical tested systems for which the two pairs of coefficients, say (*α_I_*, *β_I_*) for the system “I” and (*α_II_*, *β_II_*) for the system “II”, have been computed.

The electronegativity principles would lead with the rule that as the *α* index of a system is higher as the system will display larger reactivity/activity, here translated as growing of the antibody metabolic action; in short: “as *α* increases as grows the biological activity”, *i.e.*, the stimulation of antibody synthesis; this may be eventually called as *The First Principle of Reactive Biological Activity* (ReBiAc1).

Instead, the chemical hardness principles, especially the maximum hardness one [17], would lead with the idea that the *β* factor tendency is detrimental to the biological activity, or, analytically: “as *β* increases as slows the biological activity”, here - the immunological action; this may be eventually called as *The Second Principle of Reactive Biological Activity* (ReBiAc2).

Table 7 collects all the fitted and *reactive biological activity* (ReBiAc) data from the Figures 1–3 and the equations (1)–(9), respectively; it allows a global view of the experiments either between groups with various EF belonging to the same vaccine or not-vaccinated chickens. Furthermore, the present ReBiAc analysis finely clarifies upon the hierarchy of experiments in providing viable biological activity (here viewed as the potency of antibody synthesis). For instance, if one looks to the ordering correlation factors from the experimental systems I–IX (of Table 7) there finds the puzzling hierarchy:

(10) $$R_{IX}^{2}<R_{II}^{2}<R_{VII}^{2}<R_{V}^{2}<R_{IV}^{2}<R_{VIII}^{2}<R_{VI}^{2}<R_{III}^{2}<R_{I}^{2}$$

while this is rearranged in terms of increasing beneficial activity by the ReBiAc1 principle:

(11) $$\underset{\text{ALL}\;\text{CONTROLS}}{\underset{︸}{\alpha_{I}<\alpha_{IV}<\alpha_{VII}}}\underset{\text{UN}-\text{VAC}+\text{EF}}{\underset{︸}{<\alpha_{\text{II}}<\alpha_{\text{III}}}}\underset{\text{VAC}+\text{EF}\left(5\%\right)}{\underset{︸}{<\alpha_{V}<\alpha_{\text{VIII}}}}\underset{\text{VAC}+\text{EF}\left(15\%\right)}{\underset{︸}{<\alpha_{\text{IX}}<\alpha_{\text{VI}}}}$$

or as the decrease of antagonist activity in the light of ReBiAc2 principle:

(12) $$\underset{\text{CONTROL}-\text{VAC}}{\underset{︸}{\beta_{\text{IV}}>\beta_{\text{VII}}}}\underset{\text{UN}\text{UN}-\text{VACCINATED}}{\underset{︸}{>\beta_{\text{II}}>\beta_{I}>\beta_{\text{III}}}}\underset{\text{BIA}+\text{EF}}{\underset{︸}{>\beta_{\text{VI}}>\beta_{V}}}\underset{\text{BIAROM}+\text{EF}}{\underset{︸}{>\beta_{\text{VIII}}>\beta_{\text{IX}}}}$$

From both these chains of bio-activity orderings appears the decisive influence EF has on anti-bursal vaccines since placed on the extreme of ReBiAc principles’ records (maximums for α, and minimums for β values).

This way, the analytical and conceptual tools for cross-judging both the favourable and detrimental (natural or induced) biological actions were formulated and tested. Moreover, is phenomenologically proven that each biological system described by a parabolic dependency contains the intrinsic positive (beneficial) and negative (detrimental) effects at the metabolic level, however in different degree. Nevertheless, the actual study shows how the beneficial influence may be adjusted to prevail on the harmful one by controlling the induced biological activity, here through administrating various EF with polysaccharides from *Pleurotus*.

## 3. Experimental Section

### 3.1. Materials and methods

#### 3.1.1. Microorganisms and media

A culture of *P. ostreatus* was isolated from a mountainous district in Banat (Romania). As a result of multiple selections in the Laboratory of Plant Biotechnology, West University of Timisoara, we have obtained a high-quality offspring (a *foreign* one): MSG PL95. This strain was used in all experiments. Similarly to the case of other medicinal mushrooms (*Ganoderma lucidum* or *Grifola frondosa* or *P. pulmonarius*), we have chosen a potato extract as crop environment [18]. This environment offers many advantages regarding the quality of mycelium culture *in vitro*. The seed cultures were grown in 250 mL flasks containing 50 mL of medium containing (g·L^−1^) 20 sucrose, 3 yeast extract, 10 peptone, 0.1 NaNO_3_, 0.3 K_2_HPO_4_, 0.5 MgSO_4_ and 0.2 at 25 °C in a rotatory incubator (Gallenkamp Orbital Incubator, UK) at 120 rpm for 21 days (with the constant pH of 5.5).

#### 3.1.2. Preparation of polysaccharides extracts

The fermented broth was separated and concentrated at 40 °C to a small volume (20 mL) using a rotatory evaporator under reduced pressure (vacuum pump). Samples were filtered and then 95 and 75% ethyl alcohol (1:4, v/v) was successively added to the filtrate. The mixture of extracellular fractions (EF) was centrifuged at 7000 x *g* for 20 min to collect the precipitate, (with a yield of 35.7 g/kg, dry weight), which was dissolved in water. After further purification through ultra filtration with membrane filter (0.7 μm) pore size, molecules of more than 10^5^ Da were obtained and lyophilized. In this study, this is referred to as water-soluble EF of *P. ostreatus* with extract of polysaccharides (EPS). The contents of EPS were determined by the phenol-sulfuric acid method [19]. In the experiments, the average content of EPS in EF was as 48 mg/100 mL liquid medium.

#### 3.1.3. Animals

We have used for all experiments commercial broilers (Ross-type, 308), raised in isolation units of the Clinic for Poultry, University Science of Agriculture and Veterinary Medicine, Timişoara, Romania. The parents had been vaccinated once with IBDV live vaccine. Feed and water were provided *ad libitum.* For feeding the chickens a combined fodder has been used. The ingredients: corn, wheat, soja, monocalcium phosphate, calcium carbonate, mineral-vitamin premix, salt, plus, respectively, the analytic constituents: raw protein (min. 21.57%), raw fat (min. 4.81%), raw cellulose (max. 3.35%), raw ash (max. 2.60%), methionine (0.58%).

#### 3.1.4. The BIAVAC vaccine

The BIAVAC vaccine is a live, lyophilised, monovalent vaccine, obtained from an intermediary bursal virus stem cultivated on of non-vaccinated hen embryos. The minimum titer is 3.5 log_10_ EID_50_ (mean embryo infective dose) for each vaccine dose. The induced immunity, according to the manufacturer is developed within 8 to 14 days from date of vaccination. BIAVAC vaccine can be administered subcutaneous, in the eye, in the water intake or through aerosols. This vaccine is used at the age when the level of maternal antibodies is in obvious decrease. The vaccine is produced by the SC Pasteur Institute SA (Bucarest, Romania).

#### 3.1.5. BIAROMVAC - PA vaccine

This vaccine is prepared with the BIA–PA stem and it is used against the infectious bursal disease and does not assure complex immunity. Every vaccine dose contains the specific antigens and the protective support for lyophilisation. The specific antigen is an attenuated virus suspension of the infectious bursal disease, the BIA–PA stem, cultivated on embryonated non-vaccinated hen eggs and having a minimum titer of 3.5 log_10_ EID_50_. The protective support for lyophilisation contains a watery solution of 2% peptone and 10% lactose, i.e. one dose. The BIAROMVAC PA vaccine is used for preventing IBD in poultry-breeding districts where the infection pressure is particularly high and the disease has severe evolutions. The immunity induced by the BIAROMVAC PA vaccine develops in 2–3 weeks after the administration and reaches the optimal level after the booster doses. The duration and the immunity level are influenced by the birds’ state of health, the zoo-hygiene conditions and the interval between vaccinations. The vaccination with BIAROMVAC PA confers protection to the chickens throughout the exploitation and to the laying hens for a normal period of laying. BIAROMVAC – PA vaccine is produced and marketed by the SC Romvac Company SA (Romania).

#### 3.1.6. Experimental design

The experiment took place over a period of five weeks and we used newly hatched chickens (one day old). The parents of those chickens had been immunised with BIA (Bursal infectious aviar virus) live vaccines. So these chickens were included our experiment with a certain dose of maternal antibodies. Within the experiment, we have used 180 broiler chickens from parents 30 weeks old, immunized against bursa with live vaccine. The 180 chickens have been divided in nine groups of 20 chickens (Table 8). We have followed their maternal antibodies all along the experiment. For simplification, the groups have been named according to the treatment applied. The broiler chickens from the *Control* experimental group have received for the duration of the experiment only food and water.

In the aqueous diet of the new chickens we have administrated daily from dry weight EF, 50 and 150 mg/100 mL water (w/v, that is 5 and 15%), which correspond to experimental variants (II, III, V, VI, VIII and IX) shown in Table 8. To the broiler chickens from experimental groups IV – IX, at the end of week 1, we have stopped the water diet for eight hours, and then the vaccines have been administered in the water intake. One dose has been administered to each broiler, respectively, 3.5 log_10_ EID_50_ in the water intake. We have used for this purpose 60 doses of BIAVAC vaccine and, respectively, 60 of BIAROMVAC. We have assured a hydric diet of minimum four hours long throughout the experiment and a standard microclimate according to the age evolution for the chickens.

### 3.2. Analytical methods

#### 3.2.1. Estimation of infectious bursal disease antibodies

In order to determine the antibodies titer, we have drawn blood samples from 20 chickens every week. From vaccinated chickens, blood samples have been taken before the vaccination and on day seven of each week afterwards. The blood has been drawn from the axillary vein. The serologic examination has been made for the two vaccines by using the immunoenzymatic test Flock Check (ELISA test), using a kit of INDEXX Laboratories (USA), named Infectious Bursal Disease Virus Antibody Test Kit. The analyses have been made within the Laboratory of Virology, National Agency for Sanitary Veterinary and Food Safety, Timişoara, which is accredited by ISO quality system. The principle of the immunoenzymatic test meant to determine the relative level of anti BIA antibodies in chicken serum is the following. At the incubation of the serum diluted 1:500 (v/v) in the buckets of the micro-plate lined with viral antigen, anti-BIA antibodies from the serum (if they exist) form an antigen-antibodies complex with the antigen immobilized in the plate. After eliminating all loose material by washing, we have added the adjoint which adheres to the anti-BIA antibodies attached to the plate. The loose adjoint is eliminated by washing and then we have added the enzymatic sub layer. The color appeared as a result of the reaction is in direct proportion with the concentration of anti-BIA antibodies present in the sample. The optic density of each sample is analyzed with the help of spectrum-photometer, calibrated and verified at 650 nm, Tecan Sunrise (Tecan, Austria). The anti-BIA antibodies titer has been calculated in this respect according to the following formula:

(13) $${\text{log}}_{10}\;{\text{T}}_{\text{n}}=1.09\left({\text{log}}_{10}\frac{{\text{S}}_{\text{n}}}{{\text{P}}_{\text{n}}}+3.36\right)$$

where T_n_ = titer of antibodies, S_n_ = number of the serum sample and P_n_ = the mean of the positive controls.

If the ratio S_n_/P_n_ is superior to the value 0.2 (or the titer is higher than 396) the samples are positive and thus predict that the chickens have been vaccinated or have been exposed to the action of a virus, i.e. there is a immune response. With the help of this Infectious Bursal Disease Virus Antibody Test Kit, there can be identified even lower values of antibodies, even below the value of 396.

#### 3.2.2. Statistical analysis

The data are shown as the mean ± SD. Data were conducted to SAS (STATGRAPHICS^®^ Centurion XV, USA, 2005) for analysis of variance (ANOVA). Statistical comparison within the groups and between groups of data was carried out using the Tukey’s HSD multiple range test was used to determine significant differences (*P<0.05*) among treatments.

## 4. Conclusions

We have shown the immunomodulatory effects of EF which contain polysaccharides from *P. ostreatus* against IBDV. In the first experiment (*Control*), where we have used non-vaccinated and non-EF treated, the level of IBD maternal antibodies has decreased very fast, even from the first days of post hatching. By administering EF from *P. ostreatus* there had place a stimulation of the production of IBD antibodies, approximately to half of the value of maternal antibodies titer from the first day of hatching. In experimental groups 2 and 3, although the broilers have been vaccinated with BIAVAC and BIAROMVAC vaccine, respectively, the maternal antibodies titer has been low, the chickens being almost devoid of antibodies. By adding EF, 5 and 15%, in the water intake, as compared to the immune system reaction in experiment 1, the level of IBD antibodies has increased. This has determined us to believe that using this combination of BIAVAC and BIAROMVAC vaccine and EF from *P. ostreatus* is good for antibodies production stimulation in the period of the first days of life. In this period, according to the latest researches, it appears that the vaccines show affinity and associate with B cells, macrophages, and folliculars dendritic cells in bursa and spleen. The experiments have been rationalized by employing the parabolic chemical reactivity principles of electronegativity and chemical hardness indices to formulate and apply the ReBiAc counterparts.

## Figures and Tables

**Table 1.** The effect of different concentration of EF from *P. ostreatus* upon IBD–AB titre uppon non-vaccinated (NV) chicken in terms of average ± standard deviation (SD), and coefficient of variation (defined as the ratio: *|SD|*/*Average*).

**Experimental Source**	**Time (weeks)**	**Average ± SD**	**Coefficient of variation**
	1	5173.0^a^ ± 308.97	5.97%
Control	2	3332.05^a^ ± 260.47	7.81%
	3	754.0^a^ ± 48.11	6.38%
	4	96.0^a^ ± 19.55	20.37%
	5	0.0	

	1	5104.0^b^ ± 411.60	8.06%
NV + 5%EF	2	4532.1 b ± 545.55	12.04%
	3	1101.05 b ± 234.18	21.26%
	4	899.0 b ± 52.11	5.797%
	5	1843.0 b ± 12.51	0.67%

	1	5098.0 b ± 187.98	3.68%
NV + 15%EF	2	4166.0 b ± 426.17	10.22%
	3	2173.0 b ± 316.00	14.54%
	4	1548.85 b ± 111.37	7.19%
	5	1949.9 b ± 148.84	7.63%

a *p*<0.001,

b *p*<0.0001.

**Table 2.** The significant differences of IBD–AB titre environment between titre groups to non-vaccinated (NV) broilers and treated with EF from *P. ostreatus* at 95.0% confidence level.

**The pairs means of IBD–AB**	**Control**	**NV + 5% EF**	**NV + 15% EF**
1–2	1840.95*	571.90*	932.00*
1–3	4419.00*	4002.95*	2925.00*
1–4	5077.00*	4205.00*	3549.15*
1–5	5173.00*	3261.00*	3148.10*
2–3	2578.05*	3431.05*	1993.00*
2–4	3236.05*	3633.10*	2617.15*
2–5	3332.05*	2689.10*	2216.10*
3–4	658.00*	202.05	624.15 *
3–5	754.0*	−741.95*	223.10*
4–5	96.0	−944.00*	−401.05*

* denotes a statistically significant difference.

**Table 3.** The effect of different concentration of EF from *P. ostreatus* upon IBD–AB titre to broilers vaccinated with BIAVAC vaccine.

**Experimental Source**		**Time (weeks)**	Average*	**Standard deviation**	Coefficient of variation**
		1	3332.05	260.476	7.81%
BIAVAC		2	1157.05	111.922	9.67%
		3	185.00	35.9444	19.42%
		4	2374.00	333.598	14.05%
		5	3859.0 0	278.548	7.21%

		1	3700.00	545.832	14.75%
BIAVAC 5%EF	+	2	2097.00	395.180	18.84%
		3	2423.10	224.396	9.26%
		4	3195.05	604.461	18.91%
		5	4107.00	495.521	12.06%

		1	4166.00	426.172	10.22%
BIAVAC 15%EF	+	2	3006.05	389.239	12.94%
		3	2794.05	376.687	13.48%
		4	4155.00	587.624	14.14%
		5	5170.05	961.208	18.59%

* *p*<0.0001;

** (SD/Average).

**Table 4.** The significant differences of IBD–AB titre means between titre groups in broilers vaccinated with BIAVAC vaccine and treated with EF from *P. ostreatus* at 95.0% confidence level.

**The pairs means of IBD–AB**	**BIAVAC**	**BIAVAC + 5% EF**	**BIAVAC + 15% EF**
1–2	2175.00*	1603.00*	1159.95*
1–3	3147.05*	1276.90*	1371.95*
1–4	958.05*	504.95*	11.00
1–5	−526.95*	−407.00*	−1004.05*
2–3	972.05*	−326.10*	212.00
2–4	−1216.95*	−1098.05*	−1148.95*
2–5	−2701.95*	−2010.00*	−2164.00*
3–4	−2189.00*	−771.95*	−1360.95*
3–5	−3674.00*	−1683.90*	−2376.00*
4–5	−1485.00*	−911.95*	−1015.05*

* denotes a statistically significant difference.

**Table 5.** The effect of different concentrations of EF from *P. ostreatus* upon IBD–AB titer in chickens treated with BIAROMVAC vaccine.

**Experimental Source**	**Time (weeks)**	Average*	**Standard deviation**	**Coefficient of variation**
	1	3326.05	257.70	7.74%
BIAROMVAC	2	1361.00	258.27	18.97%
	3	217.00	45.23	20.84%
	4	2602.05	319.18	12.26%
	5	3799.05	460.48	12.12%

	1	3725.40	547.11	14.68%
BIAROMVAC + 5% EF	2	2842.00	462.71	16.28%
	3	2359.00	208.15	8.824%
	4	2982.05	334.84	11.22%
	5	3448.00	457.18	13.25%

	1	4184.70	424.25	10.13%
BIAROMVAC + 15% EF	2	2608.00	442.78	16.97%
	3	3238.00	345.98	10.68%
	4	3879.00	738.68	19.04%
	5	4161.00	553.71	13.30%

* *p*<0.0001.

**Table 6.** The significant differences of IBD–AB means titer between titer groups in broilers vaccinated with BIAROMVAC and treated with EF from *P.ostreatus* at 95.0% confidence level.

**The pairs means of IBD–AB**	**BIAROMVAC**	**BIAROMVAC + 5% EF**	**BIAROMVAC + 15% EF**
1–2	1965.05*	883.40*	1576.7*
1–3	3109.05*	1366.40*	946.7*
1–4	724.00*	743.35*	305.7
1–5	−473.00*	277.40*	23.7
2–3	1144.00*	483.00*	−630.0*
2–4	−1241.05*	−140.05	−1271.0*
2–5	−2438.05*	−606.00*	−1553.0*
3–4	−2385.05*	−623.05*	−641.0*
3–5	−3582.05*	−1089.00*	−923.0*
4–5	−1197.00*	−465.95*	−282.0

* denotes a statistically significant difference.

**Table 7.** The computed favourable and detrimental biological activity factors, α and β, according with their definitions given by Equation (9), for titers vs. weeks fitted curves displayed in Figures 1–3, employing the specific parabolic data modelled by Equation (1).

**Experimental System/Source**			**Fitted Parameters**				**Activity parameters**	

			**a**	**b**	**y_0_**	**R^2^**	α	β
UN-VAC	**I.**	Control	3676.8	386.43	8650.6	0.9809	291.04	3676.8
	**II.**	EF (5%)	3698.8	447.21	8872.8	0.8368	1671.97	3698.8
	**III.**	EF (15%)	2620.6	288.21	7678.6	0.9435	2009.74	2620.6

BIAVAC VAC	**IV.**	Control	4264.8	748.64	6740.6	0.9079	1415.39	4264.8
	**V.**	EF (5%)	2155.7	391.14	5268.8	0.8806	2689.75	2155.7
	**VI.**	EF (15%)	2222.7	423.07	5872.6	0.9309	3376.3	2222.7

BIAROM VAC	**VII.**	Control	4004	703.79	6531.4	0.8616	1540.3	4004
	**VIII.**	EF (5%)	1671.7	271.71	5097.4	0.924	2797.82	1671.7
	**IX.**	EF (15%)	1474.8	266.21	5110	0.6182	3333.62	1474.8

**Table 8.** The experimental design.

**Experimental System**		**Descriptive details**
I.	Control	NV chickens (non-vaccinated) and no EF (extracellular fraction) treatment from *Pleurotus*
II.	NV + 5% EF	NV chickens + treatment with 5% EF from *Pleurotus*
III.	NV + 15% EF	NV chickens + treatment with 15% EF from *Pleurotus*
IV.	BIAVAC Control	Vaccinated chicken with BIACAV vaccine and no EF treatment
V.	BIAVAC + 5% EF	Vaccinated chicken with BIACAV vaccine + treatment with 5% EF
VI.	BIAVAC + 15% EF	Vaccinated chicken with BIACAV vaccine + treatment with 15% EF
VII.	BIAROMVAC Control	Vaccinated chicken with BIAROMCAV vaccine and no EF treatment
VIII.	BIAROMVAC + 5% EF	Vaccinated chicken with BIAROMCAV vaccine + treatment with 5% EF
IX.	BIAROMVAC + 15%EF	Vaccinated chicken with BIAROMCAV vaccine + treatment with 15% EF
